# A One Health approach based on genomics for enhancing the *Salmonella enterica* surveillance in Colombia

**DOI:** 10.1016/j.ijregi.2023.09.008

**Published:** 2023-10-08

**Authors:** Johan F. Bernal, Paula L. Díaz, Blanca M. Perez-Sepulveda, María Fernanda Valencia-Guerrero, Viviana Clavijo, Magdalena Weisner, Lucy Angeline Montaño, Stefany A. Arevalo, Ingrid Maribel León, Luis Ricardo Castellanos, Anthony Underwood, Carolina Duarte, Silvia Argimón, Jaime Moreno, David Aanensen, Pilar Donado-Godoy

**Affiliations:** 1 Corporación Colombiana de Investigación Agropecuaria (AGROSAVIA) - C.I Tibaitata, Global Health Research Unit on antimicrobial resistance (GHRU)-AMR and Colombian Integrated Program of Antimicrobial Resistance Surveillance (COIPARS), Mosquera, Colombia; 2 Instituto Nacional de Salud (INS), Grupo de Microbiología, Bogotá DC, Colombia; 3 University of Liverpool, Institute of Infection, Veterinary & Ecological Sciences (IVES), Liverpool, United Kingdom; 4 University of the Andes, Department of Biological Sciences, Bogotá DC, Colombia; 5 Texas A&M University, Department of Veterinary Integrative Biosciences, College Station, USA; 6 Quadram Institute Bioscience, Norwich, United Kingdom; 7 University of Oxford, Global Health Research Unit (GHRU)-AMR, Big Data Institute, Oxford, United Kingdom

**Keywords:** Foodborne, *Salmonella*, WGS, Antimicrobial resistance, Virulence, One health

## Abstract

**Objectives:**

This study aimed to provide evidence of the domestic benefits of introducing an integrative genomic analysis from the One Health approach in the national surveillance of *Salmonella enterica* between 1997-2017 in Colombia.

**Methods:**

Data on *Salmonella* from clinical laboratory-based surveillance between 1997-2017 and from a national cross-sectional study at chicken retail stores in Colombia were compared using a phenotypic, molecular, and genomic approaches. Additional analysis by serovar using single nucleotide polymorphism was developed to increase the resolution of the relatedness between the interfaces.

**Results:**

Locally, the diversity and pathogenic factors of the prevalent *S. enterica* serovars associated with foodborne disease in Colombia were described using laboratory, pulse field gel electrophoresis, and whole genome sequencing data. For example, the resolution of pulse field gel electrophoresis allowed the description of two main foodborne clusters of *Salmonella Enteritidis* isolates, which were expanded to eight foodborne clades using whole genome sequencing. Likewise, virulence factors and antimicrobial resistance determinants, and mobile genetic elements that converged in the foodborne clades should be considered a public health concern in Colombia. All results by serovar were compiled in an interactive easy to share report.

**Conclusion:**

Whole genome sequencing is a technology that provides a precise assessment of emerging foodborne risks such as the *Salmonella* foodborne clades, but it requires an integrative and continued collaboration between the stakeholders across the One Health sectors to promote appropriated actions and policies in public health.

## Highlights

• Whole genome sequencing provides data that allows authorities in low and middle-income countries to better understand pathogens.
• Relatedness in *Salmonella* clades was evidence of continuous foodborne spread.
• High diversity of pathogen determinants in clades should be a public health concern.
• In total, 26% of clinically invasive *Salmonella* spp. were found to be genetically related to food isolates.

## Introduction

Over 1.7 billion cases of childhood diarrhoeal diseases occur globally every year, causing 525.000 deaths in children under 5 years old, according to the World Health Organization. Approximately half of these deaths could be linked to bacterial foodborne illnesses, especially, in low and middle-income countries (LMICs) [1], where *Salmonella* is one of the five most critical zoonotic bacteria involved in foodborne diseases associated with food production systems [2]. According to the National Public Health Institute (INS) in Colombia, *Salmonella* spp. continues to be the most important bacteria causing foodborne diseases in the country. However, in the last years, a reduction in foodborne disease reports was observed, which could be related to the COVID-19 pandemic response [3]. Recently, *Salmonella* spp. occurrence has become a public health concern in the country, due to their ability to withstand critical antimicrobials which are routinely used in clinical treatments. Colombia proposed the first integrated program in AMR surveillance in Latin America, COIPARS, and the most important outcomes showed a significant prevalence of *Salmonella* in poultry farms (41%) and retail stores (27%), and high levels of resistance to critical antibiotics such as third-generation cephalosporins (39%) and fluoroquinolones (29%) [4].

At present, the surveillance of foodborne pathogens based on whole genome sequencing (WGS) is becoming the gold standard method at the National reference laboratories. WGS has shown higher resolution results in a shorter time than traditional methods, such as pulsed-field gel electrophoresis (PFGE) [5]. An effective strategy to tackle the spread of foodborne diseases and antimicrobial resistance (AMR) has been to adopt the One Health (OH) approach, which recommends establishing a comparable and integrated information system between sectors to promote more assertive measurement strategies [6]. An OH approach of surveillance based on WGS could generate relevant integrated data on the pathogens’ dynamics across sectors [7].

Therefore, this retrospective study aimed to provide evidence of the domestic benefits of introducing an integrative genomic analysis from the OH approach in the *Salmonella* spp. national surveillance between 1997-2017 in Colombia.

## Methods

### Bacterial isolates

Initially, the molecular comparison included 801 *Salmonella* spp. isolates recovered from food and clinical between 2010-2011. Isolates from food (n = 118) belonged to the four most prevalent serovars (*S. Enteritidis*, n = 61, *S. Typhimurium*, n = 14, *S. Heidelberg*, n = 20 and *S. Paratyphi* B dT+, n = 23) recover in chicken retail stores, that included samples from 11/32 of the political divisions (departments) between October 2010 to October 2011 in Colombia [8]. On the other hand, clinical isolates were selected from the *Salmonella* national surveillance program, following the serovars previously identified in food (*S.* Enteritidis, n = 263, *S.* Typhimurium n = 416, *S.* Heidelberg n = 3 and *S.* Paratyphi B dT+ n = 1) between January 2010 and December 2011 from 28/32 public health laboratories in Colombia [9].

The genomic comparison included 811 sequences of *Salmonella* spp. isolates recovered between 1997-2017, including 62 isolates from the initial set of 118 food interface, selected based on the clonal behavior observed at the first analysis and a sample of 749 clinical *Salmonella* isolates selected from the national surveillance program based on their invasive bloodstream infection outcome. Moreover, a subset of 151 clinical sequences was defined within a cut-off of ∼ 60 single nucleotide polymorphisms (SNPs) of distance from a food isolate, as a probable median fixation rate in the core genome of *Salmonella* species in 20 years (three SNPs/year) [10].

### Phenotypic characterization

Food isolates were processed in AGROSAVIA following the procedure described by Donado-Godoy *et al.* [8]. Briefly, an aliquot of chicken carcass rinse was enriched in selective broth, incubated, and plated on XLT4 agar (Oxoid, UK). Typical *Salmonella* colonies were confirmed at serovar level following the Kauffmann-White-Le Minor scheme and the antimicrobial testing (AST) was performed by BD Phoenix TM automated microbiological system (Becton Dickinson Diagnostic Systems, Sparks, MD, USA). Interpretation of minimal inhibitory concentrations results was defined using the CLSI breakpoints to the corresponding year.

All clinical *Salmonella* isolates were confirmed in the National Reference Laboratory at INS using standard biochemical testing (Triple Sugar Iron Agar [TSI], Citrate, Urea, and motility), and Vitek II system (Biomerieux, USA). Antimicrobial susceptibility testing was performed using the Kirby-Bauer disk diffusion and minimum inhibitory concentrations (MIC) using the MicroScan autoSCAN-4-System (Beckman Coulter) according to the Clinical and Laboratory Standards Institute (CLSI) breakpoints from each year. The serovar was defined following the Kauffmann-White-Le Minor serological scheme (Difco, United States).

### Molecular characterization

All 801 isolates were processed as part of PulseNet national surveillance during 2010-2011 in both institutions. All isolates were subtyped following the Centers for Disease Control and Prevention *Escherichia coli*-*Shigella*-*Salmonella* standard PFGE protocol for *XbaI* enzyme. *Salmonella* clustering assessment including the four variables serovar, PFGE pattern (PP), location, and antimicrobial susceptibility profile was performed. The two variables serovars and PP were defined as selective measures for the similarity analysis, and binary values were assigned to location and antimicrobial susceptibility: (1) when the isolates shared the same output and (0) when the isolates did not share the same output. Pearson Similarity Coefficient (PSC) was calculated using R studio v4.2.0, and the similarity score was determined [11].

### Genomic characterization

The thermolysates of 811 *Salmonella* isolates recovered between 1997-2007 were sent to the Earlham Institute (UK) for sequencing, following the LITE pipeline, as part of the 10.000 *Salmonella* Genomes consortium, described elsewhere. All raw sequences were transferred to the national institutions and a basic QC report was provided. The Global health research unit for genomic surveillance of AMR (GHRU-AMR) pipelines (https://www.protocols.io/view/ghru-genomic-surveillance-of-antimicrobial-resista-bpn6mmhe) was built on the computational infrastructure at AGROSAVIA and used to analyzed the WGS raw data. After quality control, the raw sequences were trimmed, polished and assembled, and basic stats reports were generated as described in the Assembly GHRU-AMR pipeline v2.1.2. Multi-locus sequence typing (MLST) and AMR determinants (acquired genes and point mutations) were annotated and summary tables were generated following MLST and AMR_prediction GHRU-AMR pipelines v1.2. Plasmids and virulence factors were annotated using ARIBA (v2.14.4) with Plasmidfinder and VFDB databases, respectively. Variant calling was performed using mapping to reference genomes *Salmonella* Enteritidis str. 18569 (Genbank accession no. NZ_CP011394.1), *Salmonella* Typhimurium str. LT2 (Genbank accession no. **NC_003197.2**) and *Salmonella* Heidelberg str. SL476 (Genbank accession no. **NC_011083.1**), as described in SNP_phylogeny GHRU-AMR pipeline v1.2.2. Aligned and filtered by low quality base (%QUAL<25 || FORMAT/DP<10 || MAX(FORMAT/ADF)<2 || MAX(FORMAT/ADR)<2 || MAX(FORMAT/AD)/SUM(FORMAT/DP)<0.9 || MQ<30 || MQ0F>0.1) were performed. Phylogenetic inference was calculated using IQtree (v1.6.8) with GTR+G model and 1000 bootstrap replicates. SNPs matrix heatmap was performed using R studio (v4.2.0) with the library Complex Heatmap. Clade definition based on the SNPs matrix generated by the phylogenetic analysis was defined using rhierbaps (v1.0.1) with default settings in R studio (v4.2.2). Epidemiological, phenotypic, and genomic data were integrated in a metadata format (.csv) using R studio (v4.2.0). All results were uploaded to the Microreact platform and visualized as a public dynamic report.

### Availability of sequence data

The raw sequencing data generated from this study were deposited at the EMBL European Nucleotide Archive (ENA) repository under the project accession nos. **PRJEB35182** and **PRJEB47910**.

## Results

The integrated comparison in this study allowed to support that the dissemination of *Salmonella* between the food chain supply and humans most likely occurred for 20 years in Colombia. The analysis revealed a close genetic relation in overall *Salmonella* (1997-2017) between interfaces, displaying a long-term genetic background. These findings represent a concern for food safety in Colombia, due to the constant drip of foodborne bacteria with extended pathogenicity behaviors circulating on the food supply chain and the consumers.

The evaluation of epidemiological, phenotypical, and molecular variables (serovar, PFGE pattern, location, and antimicrobial susceptibility) evidenced a correlation across interfaces on 40.7% of the food isolates between 2010-2011. The correlation was defined as stronger (PSC = 0.87) between food and clinical isolates. In relation to genomics, the phylogenetic inference revealed that all food isolates were genetically related to previously clinical isolates between 1997-2017. The 26.3% in overall *Salmonella* spp. sequences compared in this study were clustered into 12 foodborne clades. All results by serovar were described in further detail below.

### *Salmonella* Enteritidis

#### Relatedness analysis

**Figure 1:**
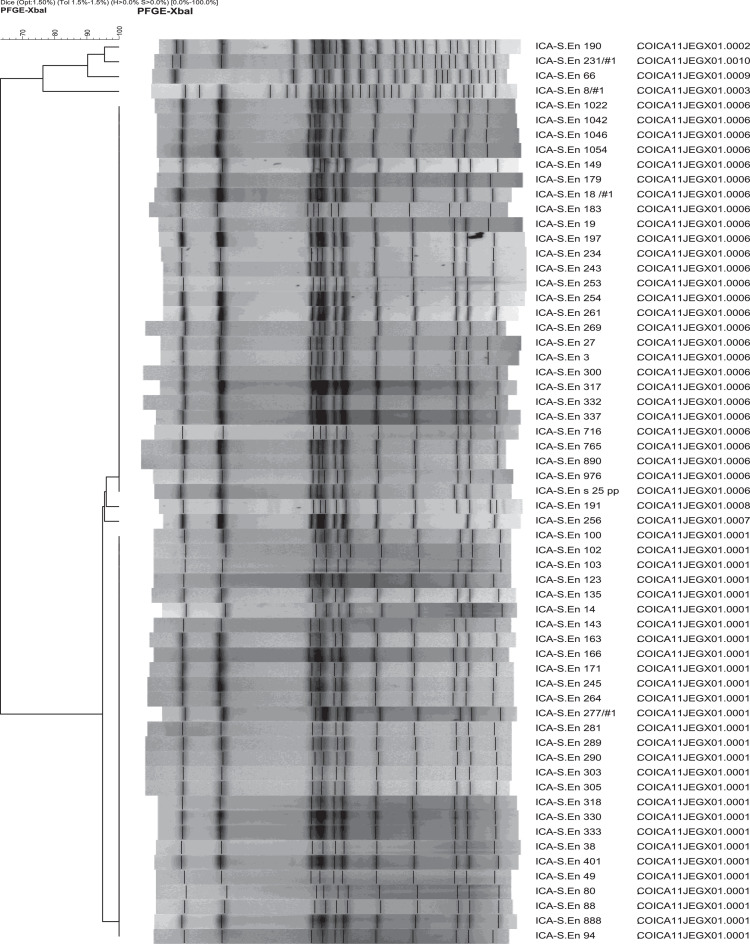
Pulsed-field gel electrophoresis clustering of *Salmonella Enteritidis* from food using DICE similarity index and clustering by UPGMA. Two main clonal clusters were identified in food isolates: Isolates with pattern ICA JEG.X01.0006 and isolates with pattern ICA JEG.X01.0001.

**Figure 2:**
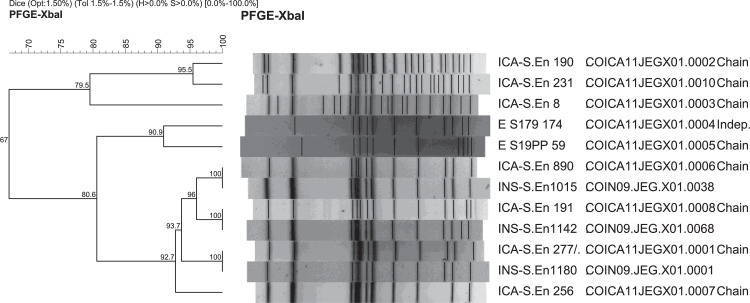
Comparison of unique PFGE patterns of *Salmonella Enteritidis* from clinical (INS) and food (ICA), using DICE similarity index and clustering by UPGMA. Three clonal relations were observed (DSI 1∙0): cluster-1 (JEG.X01.0001 - JEG.X01.0001), cluster-2 (JEG.X01.0006 - JEG.X01.0038) and cluster-3 (JEG.X01.0008 - JEG.X01.0068).

*Salmonella* Enteritidis isolates from the food interface (n = 61) showed eight unique PFGE patterns (PPs) with a DICE Similarity Index (DSI) >0.62 (Figure 1). The two most frequent food and clinical PPs exhibited clonal relatedness (DSI = 1.00; Figure 2), where the cluster-1 (INS JEG.X01.0001 - ICA JEG.X01.0001) and the cluster-2 (INS JEG.X01.0038 - ICA JEG.X01.0006) were defined. An additional cluster-3 with a food singleton PP JEG.X01.0008 that was found clonal related to clinical PP JEG.X01.0068 (Figure 2). The Integration of the four variables (serovar, PP, location, and antimicrobial susceptibility) in 61 food and 263 clinical *Salmonella* Enteritidis isolates revealed that 29.6% of the isolates were strongly correlated (PSC of 0.86; *P* <0.001) (Table S1).

PFGE, pulsed-field gel electrophoresis.

**Figure 3:**
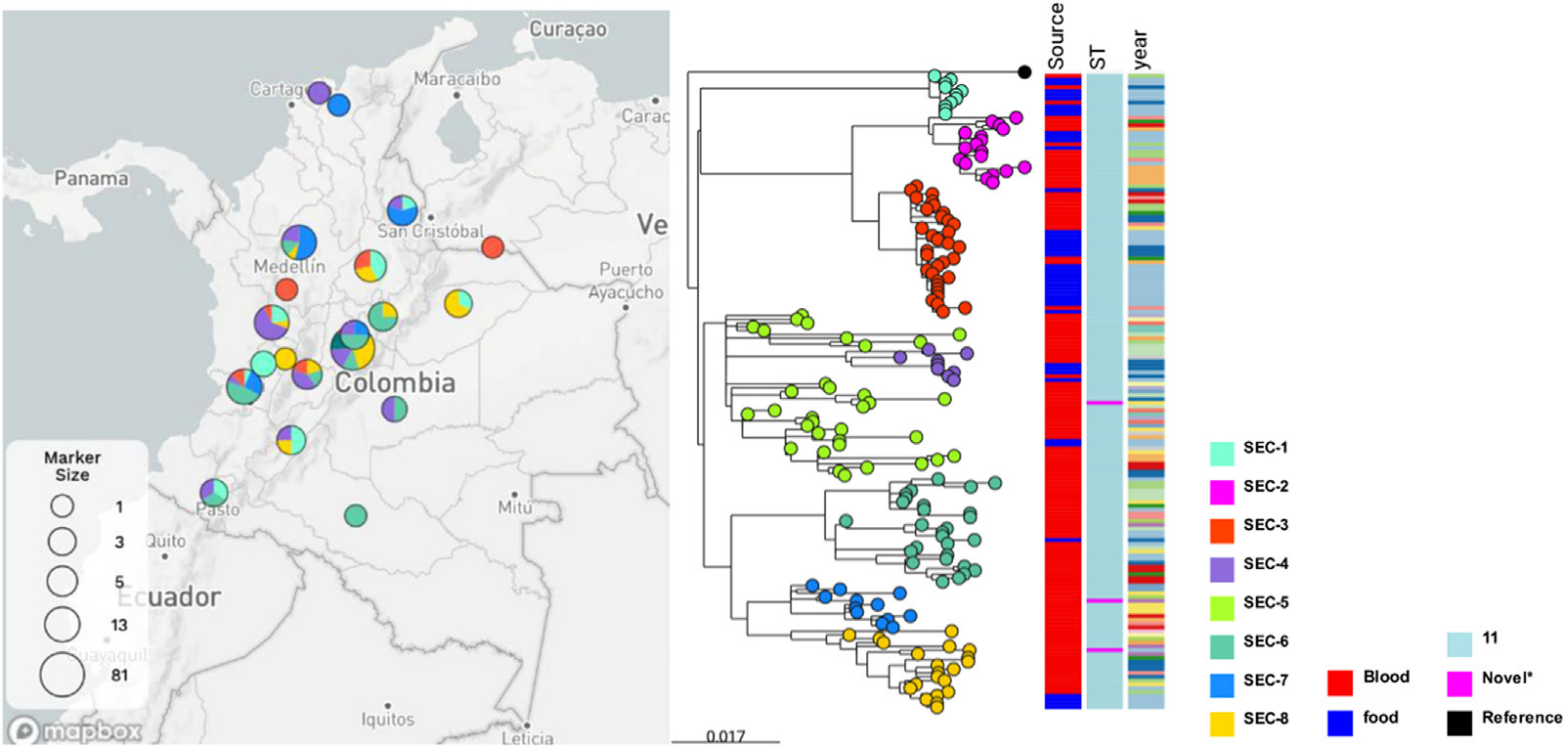
Geographical distribution and phylogeny inference of *Salmonella Enteritidis* by clade, source, and year.

SEC, *Salmonella* Enteritidis Clades; ST, sequence type.

**Figure 4:**
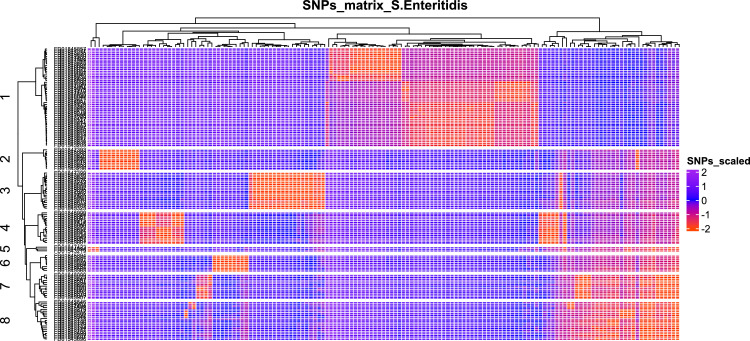
SNPs distribution matrix of *Salmonella* Enteritidis Clades.

On WGS data, an initial alignment of single nucleotide variants (SNVs) was generated mapping 394 *Salmonella* Enteritidis sequences to reference. A subset of 167 (42.4%) isolates was defined as genetically related based on SNPs distance (∼60 SNPs), including all food and 122 clinical isolates (Figure 3). Sequence types (ST) from the subset were described in the complex group (CG) 11, including ST11 (98.2%) and novel STs (1.8%). A total of eight *Salmonella* Enteritidis Clades (SECs) from 21 departments across the country were defined based on SNP loci and visualized on a phylogeny tree (Figure 4).

SNP, single nucleotide polymorphism.

#### Antimicrobial resistance

All closely related food and clinical isolates were susceptible to all antibiotics tested. The 88.1% overall *S.* Enteritidis clusters did not carry AMR-determinant genes. Moreover, twenty isolates (11.9%) from different SECs (1-8) carried AMR-determinant genes, as far as six antibiotic families. Plasmid incompatibility groups IncN_1 and IncI_1 were found in MDR clinical isolates from SECs 3, 5, and 6. Other nine plasmid incompatibility groups were observed (Table 1).

**Table 1:** Summary of *Salmonella Enteritidis* whole genome sequencing results by clades, PFGE, antimicrobial resistance, virulence, and MGE.

Serovar	Clade	# isolates	# Departmets	Range of time (years)	PFGE_Clonal_Group	ATB family	AMR genes / Mutations	Virulence genes	virulence genes related to MGE	Replicon types
S. Enteritidis	SEC-1	11	1	2010-2012	CGE-1 / CGE-2 / Others CGEs	Quinolone	qnrB81 (n=1)	TTSS-SPI-1(a), TTSS-SPI-2(a), T3SS1_effect, T3SS2_effect, T3SS1-T3SS2_effect, T8SS, fimCDFHI, bcfABCDEFG, IpfABCDE, misL, ratB, shdA, sinH, mgtB, mgtC_1, sodCI, acrA, rck, steC, slrP, sspH2, ompA_2, mig-14(b)	-	IncFIB_S (n=11) IncFII_S (n=11) Col156 (n=10) IncFII_Yp (n=3)
	SEC-2	18	7	2010-2016	CGE-1 /CGE-2	Aminoglicoside, sulfonamide, tetracycline,	aph_6 (n=1), sul2 (n=1), tetA (n=1)	TTSS-SPI-1(a), TTSS-SPI-2(a), T3SS1_effect, T3SS2_effect, T3SS1-T3SS2_effect, T8SS, fimCDFHI, bcfABCDEFG, IpfABCDE, misL, ratB, shdA, sinH, mgtB, mgtC_1, sodCI, acrA, rck, steC, slrP, sspH2, ompA_2, mig-14(b)	irp1 (n=1), ybtU (n=1)	IncFIB_S (n=18) IncFII_S (n=18) Col156 (n=18), IncFII_Yp (n=3)
	SEC-3	34	9	2009-2016	CGE-1 /CGE-2	Aminoglicoside, ß-lactam, quinolone,	aac_3_IV (n=1), aph4_la (n=1), blaCMY_2 (n=1), blaLEN_15 (n=1), qnrB19 (n=2), qnrB81 (n=1)	TTSS-SPI-1(a), TTSS-SPI-2(a), T3SS1_effect, T3SS2_effect, T3SS1-T3SS2_effect, T8SS, fimCDFHI, bcfABCDEFG, IpfABCDE, misL, ratB, shdA, sinH, mgtB, mgtC_1, sodCI, acrA, rck, steC, slrP, sspH2, ompA_2, mig-14(b)	fyuA (n=1), gogB (n=1), irp2 (n=1), ybtE (n=1), ybtP (n=1)	IncFIB_S (n=34) IncFII_S (n=34) Col156 (n=34), IncI_1 (n=2), ColpHAD28 (n=2), IncFII_Yp (n=2) ColRNAI (n=1), Col440I (n=1)
	SEC-4	9	4	2006-2011	CGE-1 /CGE-2	Aminoglycoside, ß-lactam, quinolone,	aadA1 (n=1), blaTEM_95 (n=1), qnrB82 (n=1)	TTSS-SPI-1(a), TTSS-SPI-2(a), T3SS1_effect, T3SS2_effect, T3SS1-T3SS2_effect, T8SS, fimCDFHI, bcfABCDEFG, IpfABCDE, misL, ratB, shdA, sinH, mgtB, mgtC_1, sodCI, acrA, rck, steC, slrP, sspH2, ompA_2, mig-14(b)	-	IncFIB_S (n=9), IncFII_S (n=9), Col156 (n=9), IncFII_Yp (n=2)
	SEC-5	34	11	1998-2016	CGE-1 / CGE-2 / others CGEs	Aminoglycoside, ß-lactam trimethoprim, sulfonamide, tetracycline	aph_3-lb (n=1), aph_6_ld (n=1), blaTEM_1 (n=1), dfrA14 (n=1), sul2 (n=1), tetA (n=1)	TTSS-SPI-1(a), TTSS-SPI-2(a), T3SS1_effect, T3SS2_effect, T3SS1-T3SS2_effect, T8SS, fimCDFHI, bcfABCDEFG, IpfABCDE, misL, ratB, shdA, sinH, mgtB, mgtC_1, sodCI, acrA, rck, steC, slrP, sspH2, ompA_2, mig-14(b)	-	IncFIB_S (n=34), IncFII_S (n=34), Col156 (n=34), IncFII_Yp (n=7), IncN (n=1)
	SEC-6	28	12	2004-2015	CGE-1 / CGE-2 / others CGEs	Aminoglycoside, ß-lactam trimethoprim, quinolone, sulfonamide, tetracycline, fluoroquinolone	aph_3_lb (n=1), aph_6_ld (n=1), blaTEM_1 (n=1), dfrA14 (n=1), qnrB_81 (n=1), sul2 (n=1), tet_A (n=4), gyrAD87N (n=2)	TTSS-SPI-1(a), TTSS-SPI-2(a), T3SS1_effect, T3SS2_effect, T3SS1-T3SS2_effect, T8SS, fimCDFHI, bcfABCDEFG, IpfABCDE, misL, ratB, shdA, sinH, mgtB, mgtC_1, sodCI, acrA, rck, steC, slrP, sspH2, ompA_2, mig-14(b)	-	IncFIB_S (n=28), IncFII_S (n=28), Col156 (n=28), IncFII_Yp (n=6), IncI_1 (n=2), IncN (n=1)
	SEC-7	12	7	2002-2016	CGE-1 / CGE-2 / others CGEs	Quinolone, tetracycline, fluoroquinolone	qnrB19 (n=1), tetA (n=1), gyrAD87Y (n=1)	TTSS-SPI-1(a), TTSS-SPI-2(a), T3SS1_effect, T3SS2_effect, T3SS1-T3SS2_effect, T8SS, fimCDFHI, bcfABCDEFG, IpfABCDE, misL, ratB, shdA, sinH, mgtB, mgtC_1, sodCI, acrA, rck, steC, slrP, sspH2, ompA_2, mig-14(b)	gogB (n=1)	IncFIB_S (n=12), IncFII_S (n=12), Col156 (n=12), IncX1.2 (n=6), IncFII_Yp (n=2), ColpHAD28 (n=1), Col440I (n=1)
	SEC-8	21	6	1999-2014	CGE-1 / CGE-2 / others CGEs	Aminoglicoside, ß-lactam	aph_6_ld (n=2), blaCMY_2 (n=1)	TTSS-SPI-1(a), TTSS-SPI-2(a), T3SS1_effect, T3SS2_effect, T3SS1-T3SS2_effect, T8SS, fimCDFHI, bcfABCDEFG, IpfABCDE, misL, ratB, shdA, sinH, mgtB, mgtC_1, sodCI, acrA, rck, steC, slrP, sspH2, ompA_2, mig-14(b)	-	IncFIB_S (n=21), IncFII_S (n=21), Col156 (n=20), IncFII_Yp (n=4), ColpVC (n=1), IncI_1 (n=1),

MGE, mobile genetic element; PFGE, pulsed-field gel electrophoresis; SEC, *Salmonella* Enteritidis Clades.

(a) The presence of the Salmonella pathogenicity islands are partial in some genes. (b) Virulence operons and genes were presente in all isolates, except for ompA_2 gene.

#### Virulence factors

Clinical and food close related events exhibited the same virulence repertoire across all SECs. Virulence genes related to mobile genetic elements (MGE) such as ybtE, ybtP, ybtU, fyuA, gogB, irp1, irp2, and gtrB were observed in some isolates from SECs 2, 3 and 7 (Table 1). Likewise, RND efflux system, gene acrA, and T3SS effector sspH1 were sporadically observed. Other virulence factors described previously in *Salmonella* spp. as pathogenicity islands SPI-1 and SPI-2, secretion systems III and VIII were found in more than 99.2% of the isolates, as well as, adherence, membrane, flagellar, chemotaxis kinase operons, and metal homeostasis pathways genes (Table 1).

All results of *Salmonella* Enteritidis can be visualized at https://microreact.org/project/hjw5geMmKWAAgKxhA9Y3QX-salmonellaentericaenteritidis-from-the-national-surveillance-in-colombia-as-one-health-approach).

### *Salmonella* Typhimurium

#### Relatedness analysis

*Salmonella* Typhimurium isolates from food (n = 14) showed nine unique PPs with DSI >0.72 (Figure S1). Two clusters, cluster-1 (ICA JPX.X01.0007 - INS JPX.X01.0094) and cluster-2 (ICA JPX.X01.0004 - INS JPX.X01.0197) were defined including food and clinical PPs (Figure S2). Afterwards, the four epidemiological variables from 14 food and 416 clinical clonal-related *S.* Typhimurium isolates were compared defining a strong correlation of 0.89 (*P* <0.001) in six isolates from both interfaces (Table S1).

In relation to WGS data, an initial variant calling in *S*. Typhimurium was calculated mapping to the reference a total of 267 pairs of sequences. A subset of 35 isolates found related (13.1%) was selected based on SNPs distance (∼60 SNPs): food (n = 5) and clinical (n = 30) isolates were recovered between 1999-2016 (Table S2). All sequence types were found to belong to the complex group 19 including ST's 19 (94.2%), 7423 (2.8%,) and 7617 (2.8%), geographically distributed in eight departments in the country. A total of three probable foodborne *Salmonella* Typhimurium Clades (STCs) were defined (Table S2).

#### Antimicrobial resistance

All isolates from cluster-1 and 2 showed resistance to at least one antibiotic family. The multi-drug resistance profiles were found in isolates from Bogotá belonging to cluster-2 (Table S1). Three blocks of resistance determinants genes to several antibiotics families as aminoglycoside, ß-lactam, trimethoprim, sulphonamides, macrolide, quaternary ammonium, phenicol, colistin, quinolones, and tetracycline were observed in the STCs, standing out the gene mcr-5.1 in food isolates (Table S2). The presence of plasmid incompatibility groups colpVC, colRNAI, and IncC_1 was frequently identified in isolates from STC1, likewise, IncX1.1 was observed in few isolates. All isolates from STC2 were carried IncFIIB and IncFII_S plasmids and all isolates from STC3 were carried IncQ1 and IncX1. Plasmid Col156 was also frequently observed in STC3. Co-location of qnrB gene and Col440I plasmid was related to quinolone resistance in foodborne clades (Table S2).

#### Virulence factors

MGE-related virulence genes gogB and grvA were observed in all STCs 2 and 3, and one isolates from STC 1. Otherwise, *Salmonella*´s major virulence factors as SPI-1 and SPI-2, and the secretion systems III and VIII were found in all isolates, as well as adherence, membrane, flagellar and chemotaxis kinase operons, and metal homeostasis pathways genes (Table S2).

All results of *Salmonella* Typhimurium can be explored at https://microreact.org/project/ty7n1isZqUKyu4ehFUvx1W-salmonellaenteri-catyphimurium-from-the-national-surveillance-in-colombia-as-one-health-approach.

### *Salmonella* Heidelberg

#### Relatedness analysis

*Salmonella* Heidelberg isolates from food (n = 20) showed ten unique PPs with DSI >0.69 (Figure S3). A single cluster, cluster-1 (ICA JF6.X01.0010 - INS JF6.X01.0001 – INS JF6.X01.0002) was defined (Figure S4). Four epidemiological variables from 20 food and three clinical *S.* Heidelberg isolates were compared, defining a strong correlation with a PSC of 0.73 (*P* <0.001) (Table S1).

The genomic comparison in *S*. Heidelberg was generated mapping 20 sequences to reference. A subset of 11 isolates (58%) of *S.* Heidelberg was included based on the SNPs distance (∼60 SNPs): food (n = 9) and clinical (n = 2) isolates from four departments. All STs described for this serovar belong to CG 15 including ST15 (10/11) and ST7412 (1/11) (Table S3). One *S*. Heidelberg clade (SHC) was defined including food and clinical isolates (Table S3).

#### Antimicrobial resistance

All cluster-1 isolates showed resistance to at least one antibiotic family tested. Cluster-1 closed-related isolates showed AMR profiles as CipTe (Table S1). MDR profiles were observed in two food isolates non-related to clinical interface from Santander and Bogotá. Antimicrobial resistance determinants genes to aminoglycosides, ß-lactams, trimethoprim, fosfomycin, ammonium quaternary, quinolone, streptothricin, sulphonamides, tetracycline, and some mutations associated to quinolone resistance gyrA(S83F) and parC(T57S) were observed in SHC1 isolates. Incompatibility groups IncI, IncX1.1, and IncX1.2 were found in all SHC1 isolates and the other eleven plasmid incompatibility groups were less frequent (Table S3).

#### Virulence factors

Yersinia High Pathogenicity Island (HPI) was detected in all isolates belonging to SHC1, including ybt (SXQPA) (UTE), irp1, irp2, and fyuA (Table S3). Additionally, grvA, gogB, and cdtB genes were observed occasionally in some isolates. Previously described, major virulence factors for *Salmonella* as SPI-1 and SPI-2, and secretion systems III and VIII were found in almost all SHC1 isolates, as well as adherence, membrane, flagellar and chemotaxis kinase operons, and metal homeostasis pathways genes (Table S3).

All results of *Salmonella* Heidelberg could be explored at the Microreact project (https://microreact.org/project/rwdTW9g5esRp3V6wkodz7w-salmonella-enterica-heidelberg-from-the-national-surveillance-in-colombia-as-one-health-approach).

### *Salmonella* Paratyphi B dT+

#### Relatedness analysis

*Salmonella* Paratyphi B dT+ (n = 23) from food showed thirteen unique PPs with DSI >0.53. *S.* Paratyphi B dT+ isolates were recovered from Bogotá D.C (17/23), Atlántico (3/23), Caldas (2/23) and Tolima (1/23). Comparison of PPs from human and food interfaces did not reveal any clonal or close relation between interfaces between 2010-2011, therefore, this serovar was excluded from the genomic analysis (Data shown).

#### Antimicrobial resistance

All *S.* Paratyphi B dT+ isolates recovered from food were MDR including quinolones, tetracycline, ß-lactam, ß-lactamase inhibitor, fluoroquinolone, aminoglycoside, folate pathway inhibitor, and nitrofurantoin. Two MDR profiles

AmcAmpCzoCazCroCtxXnlFoxNaCipLvxEnrStrNitTeSxt and NaSxtTeStr were observed in Atlántico and Bogotá, and Valle del Cauca, respectively.

## Conclusions

The retrospective comparison of *Salmonella* spp. from the food and clinical interfaces as proposed in this study represents the best approximation to the foodborne OH approach in Colombia. For the first time, we provided precise evidence of the relatedness of *Salmonella* food isolates obtained from poultry chain and *Salmonella* clinical isolates which are causing invasive disease, especially, in childhood and elders for a long period of time and a wide geographical distribution in Colombia. Likewise, the early and precise detection of emerging foodborne risks, as the convergency of multi-drug resistance and high virulence observed in foodborne clades in this study should be a public health priority [12].

WGS has become the gold standard procedure for foodborne surveillance in public health laboratories in High-Income countries, showing huge advantages and best resolution on investigations of pathogens [13]. The COVID-19 pandemic provides a unique opportunity to sensibilize general audiences, decisionmakers and policymakers in LMICs about the applicability of WGS on infectious disease surveillance and monitoring. Foodborne study in USA showed that WGS reduced 34.0% of the cases misplaced in outbreaks, revealing a higher percentage of confirmed outbreak cases by WGS (78.0%) than PFGE (46.0%) [14]. This study adds evidence of the benefits of WGS over PFGE on *Salmonella* spp. surveillance and outbreak investigations across interfaces, resolving close genetic relations up to three-fold more discriminatory in the same population than PFGE, and with great additional pathogenic data. Nevertheless, the implementation of genomics in LMICs has multi-factor challenges, especially high cost, complexity, infrastructure, and scarce trained human resources, which need to be considered for the genomic surveillance implementation domestically [15]. Similarly, the cost of maintenance of the integrated surveillance system of foodborne bacteria also remains one the principal barriers in LMICs, nevertheless, multi-lateral initiatives such as the National Action Plans in AMR provided countries, a roadmap to obtain legal framework, sustainability, inter-sectorial commitment, agreements, governance and policies for the establishment of new technologies as WGS in health systems [16]. Other WGS challenges are the interpretation and communication of results between stakeholders and decisionmakers, that is not as straight-forward as other typing methods that have a stable nomenclature [14]. Nonetheless, the easy share interactive WGS reports used in this study could help to overcome these challenges in the national reference laboratories in Colombia and elsewhere [17].

Application of WGS to *Salmonella* Enteritidis, the most prevalent serovar in Colombia, allowed us to understand the limitations of current molecular surveillance (PFGE), resolving close genetic relations across the interfaces. The SECs associated with poultry in this study were more diverse than other similar studies, suggesting a higher complexity in the contamination sources in Colombia [18]. Moreover, the three foodborne STCs identified in this study exhibited clonal dissemination of the virulence factors, AMR determinants, and mobile platforms, which support the relevance of this serovar as foodborne in Colombia. Nevertheless, *S.* Typhimurium outbreaks are frequently related to certain types of food chains such as swine and cattle, more than poultry, and due to the lower number of food isolates described in this study, the presence in poultry meat could be related to cross-contamination at retail stores as other raw meats, human manipulation, equipment or transport [19].

Nonetheless, WGS provided specific biomarkers in foodborne clades that could be used in surveillance and monitoring programs on less expensive routine polymerase chain reaction tracing or rapid detection methods, especially in LMICs [20]. An example of how WGS data could address dynamic surveillance of foodborne emerging risks in the populations is the presence of colistin resistance gene mcr-5.1, first reported in Colombia, that had been gaining prominence on antimicrobial control and surveillance since Colombia had been declared as carbapenem resistance endemic country and colistin is the last clinical treatment resource [21].

Remarkably, WGS also allowed us to observe in the *Salmonella* Heidelberg clade, the convergency of complex AMR profiles and virulence factors as Yersiniabactins located in the *Yersinia* High Pathogenicity Island (HPI). In 2021, HPI was described for the first time in *Salmonella* serovar Heidelberg ST-15 in Brazil. Previously, HPI was described in other *Salmonella* serovars in clinical and animal production interfaces in Germany and USA [22]. Similar reports in E. coli have been related HPI with the increasing virulence and resilience in the host, besides increasing pathogenicity in poultry production systems [22]. In Colombia, *S.* Heidelberg has been reported as the second most prevalent serovar in poultry production, but less common in human-invasive infections [9]. Nevertheless, this serovar should be monitored in the national surveillance due to the pathogenic potential and the relevance as invasive bacteria in other countries [23]. Similarly, *S.* Paratyphi B dT+ (Java) has been reported as the most prevalent serovar recovered at poultry farms, slaughterhouses, and retail stores in Colombia, despite this, the clinical isolates were scarce in this study [24]. Nonetheless, it should be monitored as a public health concern in Colombia, due to its high performance as multi-drug resistance reservoir and the increasing of paratyphoid infectious reports at the clinical interface [4].

Although *Salmonella* from the clinical was defined with the highest complexity of AMR profiles, suggesting higher selective pressures at clinical and community, *Salmonella* from poultry raw meat was found as the primary carrier of acquired virulence factors [25,26]. Food isolates were more frequently clustered in Clade 1 y 3. Otherwise, additional food sources should be involved in human-invasive Salmonellosis infections in Colombia, since this study explained just a quarter of the invasive samples. Therefore, it is essential to provide regular WGS data from the other food chains, also from other interfaces as environmental to generate a much more comprehensive analysis of *Salmonella* dissemination and its zoonotic dynamics in Colombia [27], [28], [29].

Finally, the precise data resulting from WGS allows authorities to follow most certainly the establishment of risks and relatedness in foodborne, especially, in pathogens with clonal behaviors such as *Salmonella* Enteritidis [30]. Therefore, having integrated data and a powerful toolbox for the certain assessment of genetic relations across interfaces can help to establish dissemination routes, support real-time measurements and control actions, and address food policies in public health.

## Declarations of competing interest

The authors have no competing interests to declare.

## Appendix Supplementary materials

Figure S1. PFGE clustering of *Salmonella Typhimurium* from food samples using DICE similarity index and clustering by UPGMA. Three clonal-related clusters were identified. Image, application 1

Figure S2. Comparison of unique *Salmonella Typhimurium* PFGE patterns from clinical (INS) and food (ICA) using DICE similarity index and clustering by UPGMA. Two clonal clusters were found (SI%:100): Cluster-1 JPX.X01.0007 - JPX.X01.0094 and cluster-2 JPX.X01.0004 - JPX.X01.0197 Image, application 2

Figure S3. PFGE clustering of *Salmonella Heidelberg* from food samples using DICE similarity index and clustering by UPGMA. Three clonal-related clusters were identified. Image, application 3

Figure S4. Comparison of unique *Salmonella Heidelberg* PFGE patterns from clinical and food using DICE similarity index and clustering by UPGMA. One clonal cluster was found (DSI%=100): Cluster-1 COIN09.JF6.X01.0001 - COICA11.JFG.X01.0010. Another clinical pattern was found close related (DSI%=97) to the clonal cluster-1: COIN09.JF6.X01.0002 Image, application 4

Table S1. Similarity analysis of *Salmonella* spp. isolates from food and clinical compared by serovar, PFGE profile, Location, and antimicrobial resistance (AMR). Table S2. Summary of *Salmonella Typhimurium* WGS results by clades, antimicrobial resistance, virulence, and MGE. Table S3. Summary of *Salmonella Heidelberg* WGS results by clades, antimicrobial resistance, virulence, and MGE. Image, application 5
