# Supplementary material for: A One Health approach based on genomics for enhancing the Salmonella enterica surveillance in Colombia
Source: IJID Reg. 2023 Oct 8;9:80–7. doi: 10.1016/j.ijregi.2023.09.008 (PMC10630622; doi:10.1016/j.ijregi.2023.09.008)
Supplement: Supplementary file 1 — Figure S1. PFGE clustering of Salmonella Typhimurium from food samples using DICE similarity index and clustering by UPGMA. Three clonal-related clusters were identified. [file mmc1.pdf]

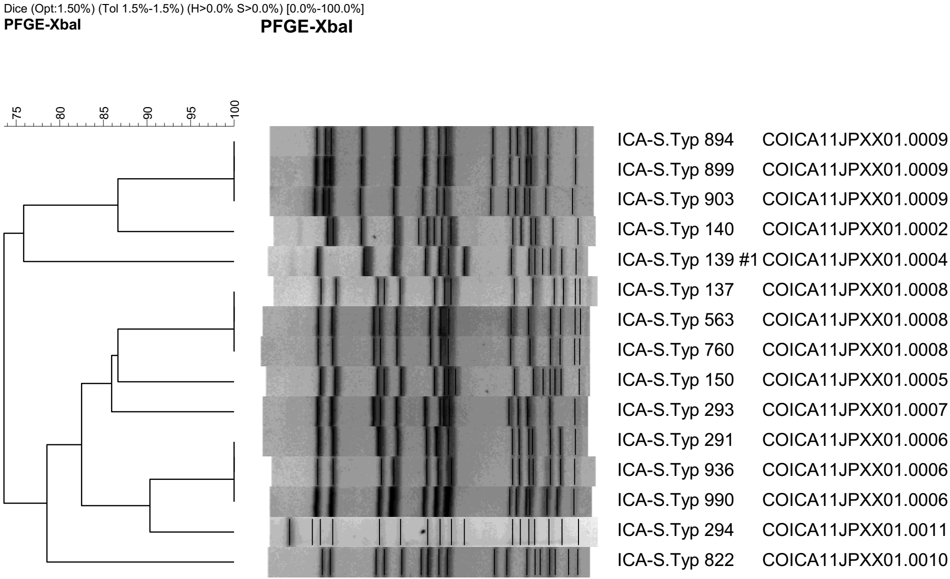
Dice (Opt:1.50%) (Tol 1.5%-1.5%) (H>0.0% S>0.0%) [0.0%-100.0%]
PFGE-XbaI
PFGE-XbaI
75
80
85
90
95
100
ICA-S.Typ 894 COICA11JPXX01.0009
ICA-S.Typ 899 COICA11JPXX01.0009
ICA-S.Typ 903 COICA11JPXX01.0009
ICA-S.Typ 140 COICA11JPXX01.0002
ICA-S.Typ 139 #1 COICA11JPXX01.0004
ICA-S.Typ 137 COICA11JPXX01.0008
ICA-S.Typ 563 COICA11JPXX01.0008
ICA-S.Typ 760 COICA11JPXX01.0008
ICA-S.Typ 150 COICA11JPXX01.0005
ICA-S.Typ 293 COICA11JPXX01.0007
ICA-S.Typ 291 COICA11JPXX01.0006
ICA-S.Typ 936 COICA11JPXX01.0006
ICA-S.Typ 990 COICA11JPXX01.0006
ICA-S.Typ 294 COICA11JPXX01.0011
ICA-S.Typ 822 COICA11JPXX01.0010
