# Supplementary material for: A One Health approach based on genomics for enhancing the Salmonella enterica surveillance in Colombia
Source: IJID Reg. 2023 Oct 8;9:80–7. doi: 10.1016/j.ijregi.2023.09.008 (PMC10630622; doi:10.1016/j.ijregi.2023.09.008)
Supplement: Supplementary file 2 — Figure S2. Comparison of unique Salmonella Typhimurium PFGE patterns from clinical (INS) and food (ICA) using DICE similarity index and clustering by UPGMA. Two clonal clusters were found (SI%:100): Cluster-1 JPX.X01.0007 - JPX.X01.0094 and cluster-2 JPX.X01.0004 - JPX.X01.0197 [file mmc2.pdf]

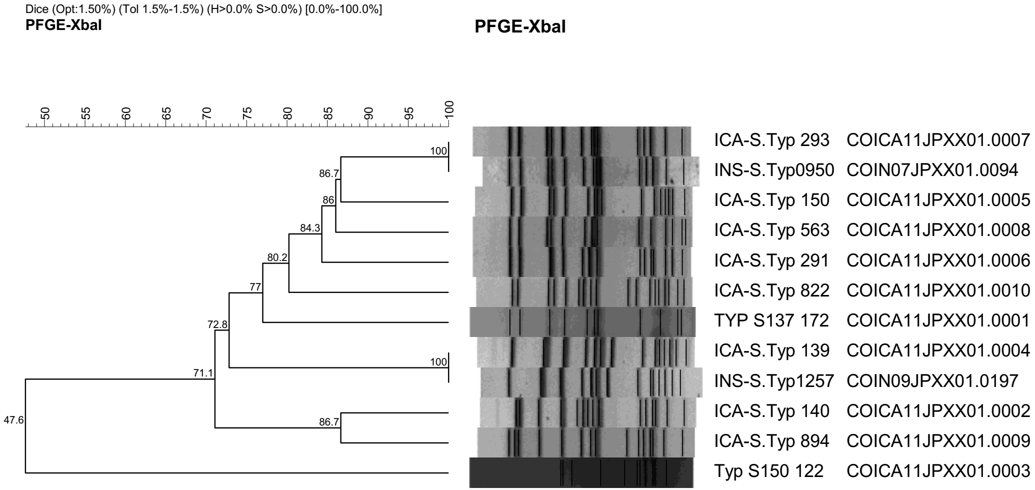

Dice (Opt:1.50%) (Tol 1.5%-1.5%) (H>0.0% S>0.0%) [0.0%-100.0%]
PFGE-XbaI
PFGE-XbaI
50
55
60
65
70
75
80
85
90
95
100
100
86.7
86
84.3
80.2
77
72.8
100
71.1
47.6
86.7
ICA-S.Typ 293 COICA11JPXX01.0007
INS-S.Typ0950 COIN07JPXX01.0094
ICA-S.Typ 150 COICA11JPXX01.0005
ICA-S.Typ 563 COICA11JPXX01.0008
ICA-S.Typ 291 COICA11JPXX01.0006
ICA-S.Typ 822 COICA11JPXX01.0010
TYP S137 172 COICA11JPXX01.0001
ICA-S.Typ 139 COICA11JPXX01.0004
INS-S.Typ1257 COIN09JPXX01.0197
ICA-S.Typ 140 COICA11JPXX01.0002
ICA-S.Typ 894 COICA11JPXX01.0009
Typ S150 122 COICA11JPXX01.0003
