# Supplementary material for: A One Health approach based on genomics for enhancing the Salmonella enterica surveillance in Colombia
Source: IJID Reg. 2023 Oct 8;9:80–7. doi: 10.1016/j.ijregi.2023.09.008 (PMC10630622; doi:10.1016/j.ijregi.2023.09.008)
Supplement: Supplementary file 3 — Figure S3. PFGE clustering of Salmonella Heidelberg from food samples using DICE similarity index and clustering by UPGMA. Three clonal-related clusters were identified. [file mmc3.pdf]

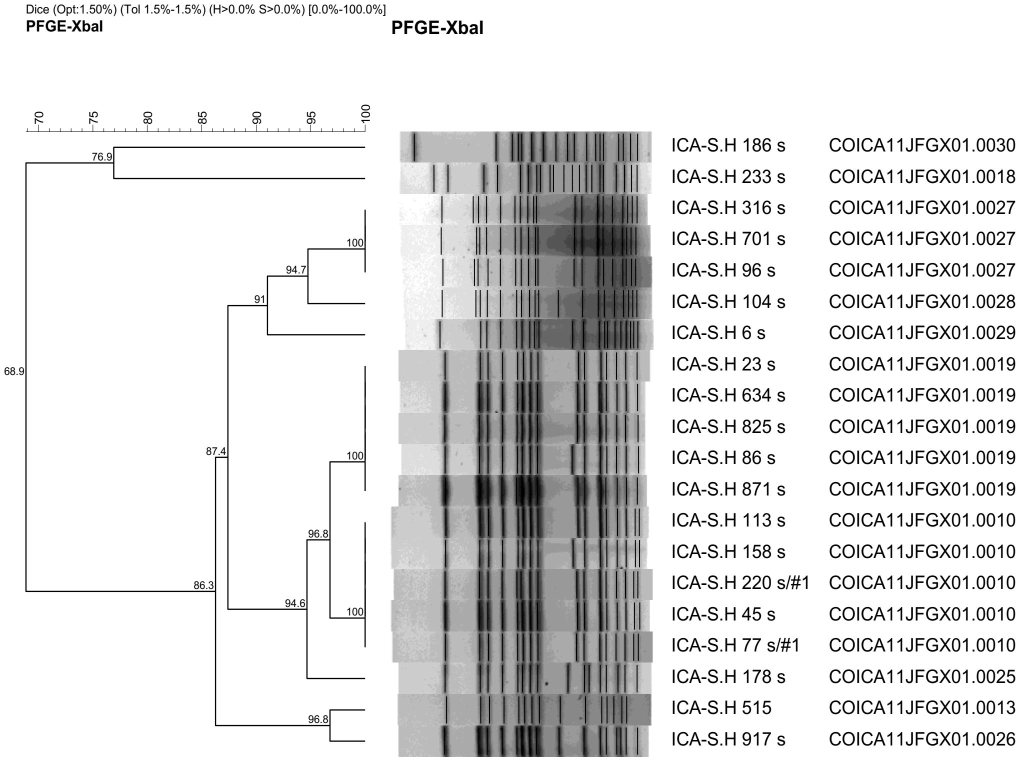
Dice (Opt:1.50%) (Tol 1.5%-1.5%) (H>0.0% S>0.0%) [0.0%-100.0%]
PFGE-XbaI
PFGE-XbaI
70
75
80
85
90
95
100
76.9
68.9
100
94.7
91
87.4
100
96.8
86.3
94.6
100
96.8
ICA-S.H 186 s COICA11JFGX01.0030
ICA-S.H 233 s COICA11JFGX01.0018
ICA-S.H 316 s COICA11JFGX01.0027
ICA-S.H 701 s COICA11JFGX01.0027
ICA-S.H 96 s COICA11JFGX01.0027
ICA-S.H 104 s COICA11JFGX01.0028
ICA-S.H 6 s COICA11JFGX01.0029
ICA-S.H 23 s COICA11JFGX01.0019
ICA-S.H 634 s COICA11JFGX01.0019
ICA-S.H 825 s COICA11JFGX01.0019
ICA-S.H 86 s COICA11JFGX01.0019
ICA-S.H 871 s COICA11JFGX01.0019
ICA-S.H 113 s COICA11JFGX01.0010
ICA-S.H 158 s COICA11JFGX01.0010
ICA-S.H 220 s/#1 COICA11JFGX01.0010
ICA-S.H 45 s COICA11JFGX01.0010
ICA-S.H 77 s/#1 COICA11JFGX01.0010
ICA-S.H 178 s COICA11JFGX01.0025
ICA-S.H 515 COICA11JFGX01.0013
ICA-S.H 917 s COICA11JFGX01.0026
