# Supplementary material for: A One Health approach based on genomics for enhancing the Salmonella enterica surveillance in Colombia
Source: IJID Reg. 2023 Oct 8;9:80–7. doi: 10.1016/j.ijregi.2023.09.008 (PMC10630622; doi:10.1016/j.ijregi.2023.09.008)
Supplement: Supplementary file 4 — Figure S4. Comparison of unique Salmonella Heidelberg PFGE patterns from clinical and food using DICE similarity index and clustering by UPGMA. One clonal cluster was found (DSI%=100): Cluster-1 COIN09.JF6.X01.0001 - COICA11.JFG.X01.0010. Another clinical pattern was found close related (DSI%=97) to the clonal cluster-1: COIN09.JF6.X01.0002 [file mmc4.pdf]

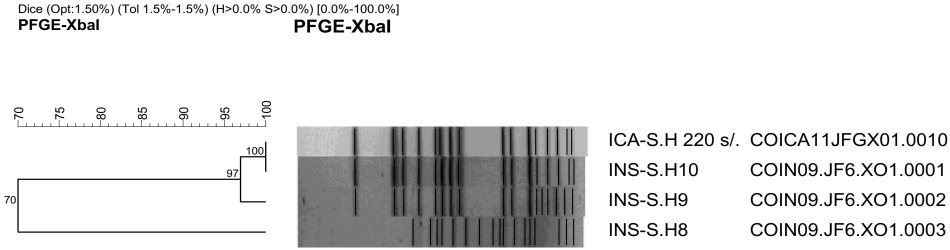
Dice (Opt:1.50%) (Tol 1.5%-1.5%) (H>0.0% S>0.0%) [0.0%-100.0%]
PFGE-XbaI
PFGE-XbaI
70
75
80
85
90
95
100
100
97
70
ICA-S.H 220 s/.
COICA11JFGX01.0010
INS-S.H10
COIN09.JF6.XO1.0001
INS-S.H9
COIN09.JF6.XO1.0002
INS-S.H8
COIN09.JF6.XO1.0003
